# Enhanced tolerance to drought stress resulting from *Caragana korshinskii* CkWRKY33 in transgenic *Arabidopsis thaliana*

**DOI:** 10.1186/s12863-021-00965-4

**Published:** 2021-03-10

**Authors:** Zhen Li, Fengping Liang, Tianbao Zhang, Na Fu, Xinwu Pei, Yan Long

**Affiliations:** 1 Biotechnology Research Institute, Chinese Academy of Agricultural Sciences, Beijing, 100081 China; 2 Ministry of Education Key Laboratory for Ecology of Tropical Islands, College of Life Sciences, Hainan Normal University, Haikou, 571158 China

**Keywords:** CkWRKY33, WRKY, Transgenic *Arabidopsis thaliana*, Drought stress

## Abstract

**Background:**

It is well known that WRKY transcription factors play important roles in plant growth and development, defense regulation and stress responses.

**Results:**

In this study, a WRKY transcription factor, WRKY33, was cloned from *Caragana korshinskii*. A sequence structure analysis showed that it belonged to the Group-I type. Subcellular localization experiments in tobacco epidermal cells showed the presence of CkWRKY33 in the nucleus. Additionally, *CkWRKY33* was overexpressed in *Arabidopsis thaliana*. A phenotypic investigation revealed that compared with wild-type plants, *CkWRKY33*-overexpressing transgenic plants had higher survival rates, as well as relative soluble sugar, proline and peroxidase contents, but lower malondialdehyde contents, following a drought stress treatment.

**Conclusions:**

This suggested that the overexpression of *CkWRKY33* led to an enhanced drought-stress tolerance in transgenic *A. thaliana*. Thus, CkWRKY33 may act as a positive regulator involved in the drought-stress responses in *Caragana korshinskii*.

**Supplementary Information:**

The online version contains supplementary material available at 10.1186/s12863-021-00965-4.

## Background

Plants undergo different kinds of environmental stresses, such as exposure to drought, salt, cold and others during their whole life cycles [1]. Stresses usually affect plant growth, survival and yield. Plants have developed diverse adaptive mechanisms to respond to various abiotic stresses during the long-term evolutionary process [2]. Most of these mechanisms are controlled by networks regulated by transcription factors (TFs) [3]. TFs are proteins that can specifically bind to cis-acting elements, and regulate the expression of downstream target genes [4].

WRKY TFs, which are named for their highly conserved WRKY domains, form a large family in higher plants and play important roles in many physiological processes [5, 6]. Many WRKY TFs have been discovered in various plants. For example, 74 WRKY members exist in the Arabidopsis genome, 109 in the rice genome, 57 in the cucumber genome, 105 in the willow genome, and 46 in the rape genome [6–10]. On the basis of the number of WRKY domains and the structural characteristics of zinc fingers, all the members of the WRKY TF family are divided into three categories, I, II and III [5]. Group-I members generally contain two WRKY domains in the N-and C-terminal end, and its zinc finger structure type is C2H2 (cx4–5-c-× 22–23-h-× 1-h). Members include such as AtWRKY54, AcWRKY9 and OsWRKY96. Group-II members contain only one WRKY domain structure, and the structure of the zinc finger is the same as in Group-I. Members include such as GmWRKY21, AtWRKY40 and AcWRKY3. Most of the WRKY TFs belong to this type. Group–III members contain only one WRKY domain structure, and the zinc finger structure is the C2-HC (C-X7-C-X23-H-X1-C) type. Members include such as AtWRKY4, AtWRKY54 and VlWRKY48 [11, 12].

WRKYs are involved in the drought-stress responses of plants. For example, *WRKY54* and *WRKY70* negatively regulate osmotic stress in Arabidopsis, and these two genes are involved in the regulation of plant growth and response to drought [13, 14]. The overexpression of *OsWRKY11* under the control of the HSP101 promoter leads to the enhancement of drought resistance, which is manifested as slower leaf withering and higher survival rates of green plants [15]. The over-expression of *OsWRKY45* and *OsWRKY72* can change the drought tolerance of Arabidopsis plants, which may be related to the induction of abscisic acid/stress-related genes [16, 17]. The knockout mutant *oswrky47* was highly sensitive to drought, resulting in decreased yield, while the over-expressed mutant of *OsWRKY47* was more resistant to drought [18]. A transcriptome analysis showed that the expression levels of *WRKY16*, *WRKY59* and *WRKY61* are up-regulated after drought treatments in common wheat, and these genes may participate in drought-stress response [19].

*Caragana korshinskii* Kom. is a leguminous shrub that is widely distributed across desert habitats with gravel-like, sandy, and saline soils in Asia and Africa. *C. korshinskii* has highly developed roots and a strong tolerance to abiotic stress [20]. Studies on *C. korshinskii* have mainly focused on biological characteristics, physiological changes and anatomical structure [21]. Relatively few drought-related genes have been identified in *C. korshinskii*, such as *CkLEA1* [22], *CkWRKY1* [23] and *CKNCED1* [24]. Most of the genes were cloned using PCR-based methods without any gene function analyses. In our previous study, we used RNA-Seq and a de novo assembly method to produce a transcriptome library of *C. korshinskii* Kom [25]. Then, we identified the drought-resistance genes by comparing two digital gene expression libraries, and several drought-related genes have been identified. Based on the bioinformatics analysis, here, we cloned the *WRKY33* gene and analyzed its gene structure and type in the *C. korshinskii* genome. Then, the drought-resistant phenotypes and physiological indices of the Arabidopsis transgenic plants were determined to verify the *CkWRKY33* gene’s function, which could play an important role in *C. korshinskii* growing under drought-stress conditions.

## Results

### *CkWRKY33* cloning and sequence analysis

In our previous study, we used 1 month old seedling of *C. korshinskii* Kom. to do drought treatment, and then we did RNA-seq and de novo assembly (BioSample: SAMN03121496). The results showed that there were 440 differentially expressed genes (DEGs) between drought and control plants, and among the DEGs, 39 unigenes showed up-regulated expression after drought treatment. After comparing with the database, we named one unigene, *com66203* as *CkWRKY33*.

**Fig. 1:**
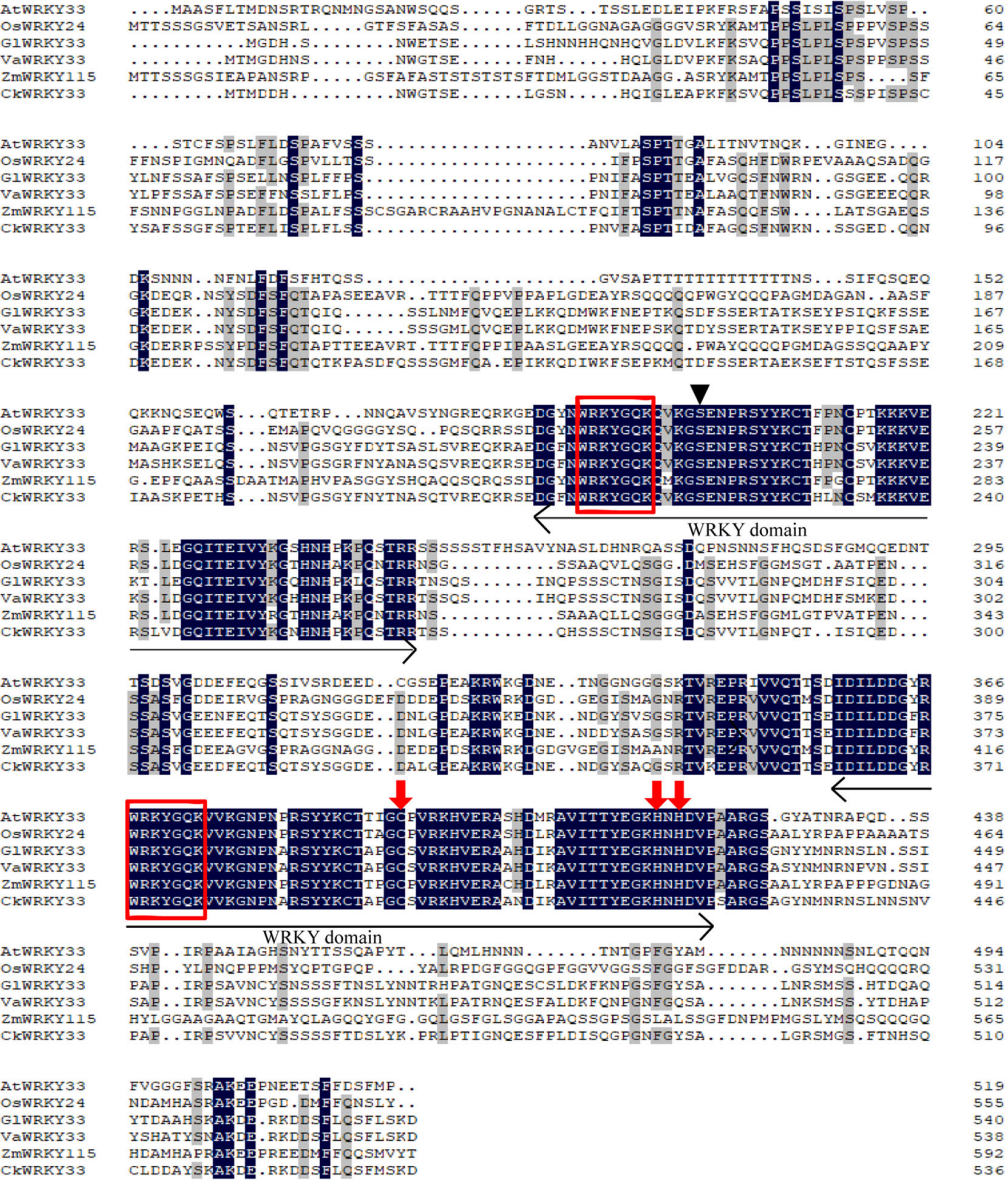
Alignment of the putative amino acid sequence of CkWRKY33 with sequences from Arabidopsis (ACE74719.1), *Glycine max* (XP_014626730.1), rice (Os01t0826400), *Vigna angularis* (XP_017442339.1), and maize (Zm00001d012482_T001). Identical amino acids are shaded in black. Approximately 60 amino acids of the WRKY domain and the cysteine and histidine residues of the putative zinc-finger motif are marked by a two-headed arrow and red arrow, respectively. The putative nuclear localization signal and the highly conserved amino acid sequence WRKYGQK in the WRKY domain are enclosed by red boxes

The full-length cDNA of *CkWRKY33* was obtained from total RNA extracted from drought-stressed *C. korshinskii* Kom. leaves using RT-PCR. The nucleotide sequence of the *CkWRKY33* gene is 2075 bp in length, consisting of a 23-bp 5′ untranslated region, an 1614 bp open reading frame (ORF) and a 345-bp 3′ UTR. The ORF encodes a putative 537-amino acid protein. Sequence alignments between CkWRKY33 and other plant WRKY proteins indicated that the amino acid sequences of these proteins share a high similarity. The sequence identity between CkWRKY33 and the other proteins in the analysis ranged from 39 to 85% (Fig. 1). A multiple sequence alignment analysis revealed that CkWRKY33 contains two putative WRKY domains followed by a C2H2-type zinc-finger motif, a putative nuclear localization signal and a short conserved structural motif (C-motif), indicating that CkWRKY33 belongs to Group-I of the WRKY family (Fig. 1).

**Fig. 2:**
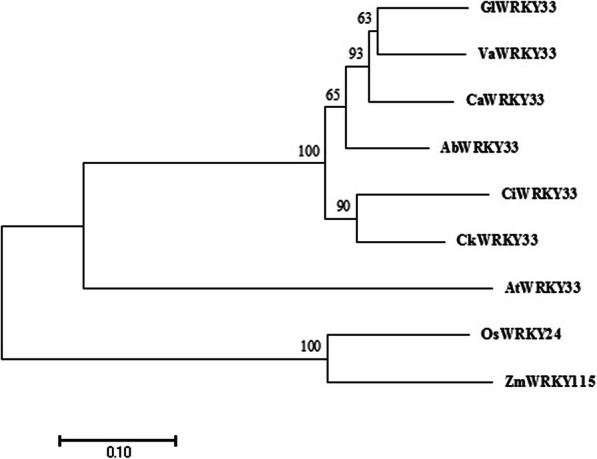
Phylogenetic analysis of CkWRKY33 and closely related WRKY transcription factors from other species. The accession numbers of selected WRKYs are as follows: Arabidopsis (ACE74719.1), *Glycine max* (XP_014626730.1), rice (Os01t0826400), *Vigna angularis* (XP_017442339.1), maize (Zm00001d012482_T001), *Cajanus cajan* (XP_020234989.1), *Cicer arietinum* (XP_004492519.1) and *Abrus precatorius* (XP_027352741.1)

A phylogenetic tree was constructed to investigate the evolutionary relationships among CkWRKY33 and other WRKY proteins. As shown in Fig. 2, CkWRKY33 showed a close relationship with AtWRKY33 in Arabidopsis, WRKY24 in rice and WRKY115 in maize. These proteins participate in plant response to abiotic stresses [26, 27]. Thus, these proteins having high homology levels among different species, may share some similar functions.

### Subcellular localization of CkWRKY33

**Fig. 3:**
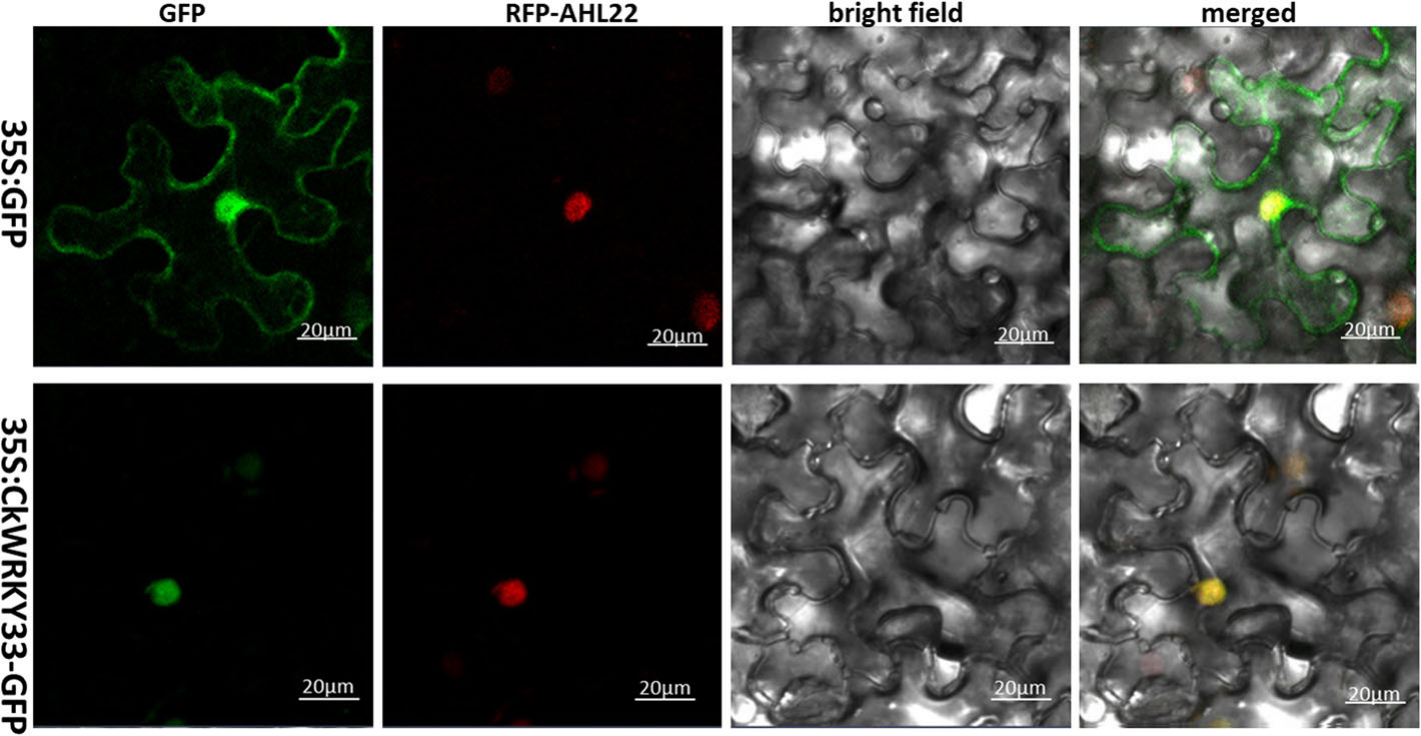
Nuclear localization of CkWRKY33. The 35 s-CkWRKY33-GFP fusion protein and GFP alone which were driven by the CaMV35S promoter, were transiently expressed in tobacco epidermal cells and visualized by fluorescence microscopy

To determine the subcellular localization of CkWRKY33, the ORF of CkWRKY33 without the termination codon was fused to the 5′ end of the GFP reporter gene under the control of the CaMV35S promoter. The recombinant construct and the GFP vector were independently introduced into tobacco epidermal cells. Confocal imaging showed that the 35 s-CkWRKY33-GFP fusion protein was exclusively localized in the nuclear. By contrast, tobacco epidermal cells transformed with the 35 s-GFP vector alone displayed fluorescence throughout the entire cell, demonstrating that CkWRKY33 is a nuclear localized protein (Fig. 3).

### Overexpression of *CkWRKY33* enhances tolerance to mannitol stress

**Fig. 4:**
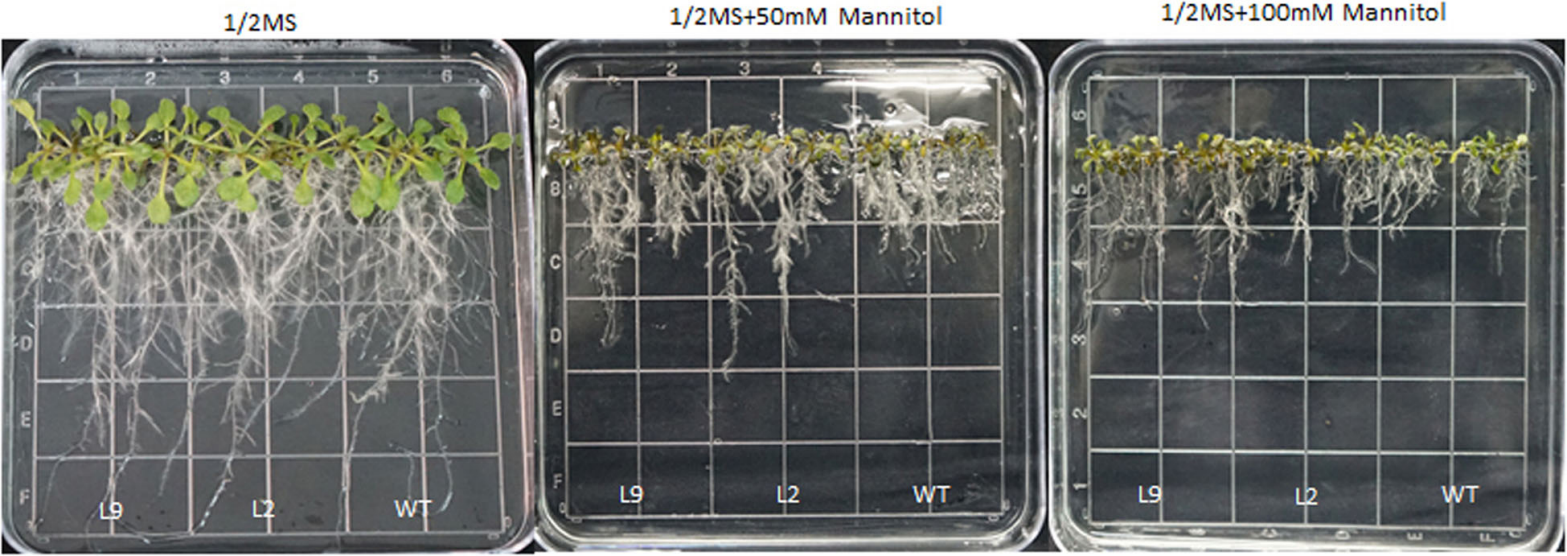
Effects of mannitol stress on the plant growth of wild-type (WT) and *CkWRKY33* transgenic Arabidopsis lines. Seedlings at 15 d after transfer to 1/2MS, 1/2MS + 50 mM mannitol, and 1/2MS + 100 mM mannitol

The full-length cDNA of *CkWRKY33* under the control of the CaMV35S promoter was transformed into Arabidopsis. After positive transformants were screened and self-crossed, the seeds of T_2_ transgenic homozygous and wild-type (WT) lines were sown on normal 1/2MS medium and 1/2MS medium supplemented with mannitol. On 1/2MS solid medium, the growth of transgenic lines was generally similar to that of WT, with no obvious change in root length. Under mannitol-treatment conditions, the seedlings of both the transgenic and WT plants grew weakly, the rosette leaves turned yellow, and the root lengths became shorter as the mannitol concentration increased. With both 50 mM and 100 mM mannitol treatments, the root lengths of transgenic plants were longer than those of WT plants (Fig. 4). Thus, the root length of Arabidopsis was changed by mannitol stress, and the *CkWRKY33* gene may have effect on the mannitol resistance of the plant.

### Overexpression of *CkWRKY33* enhances the tolerance to drought stresses

**Fig. 5:**
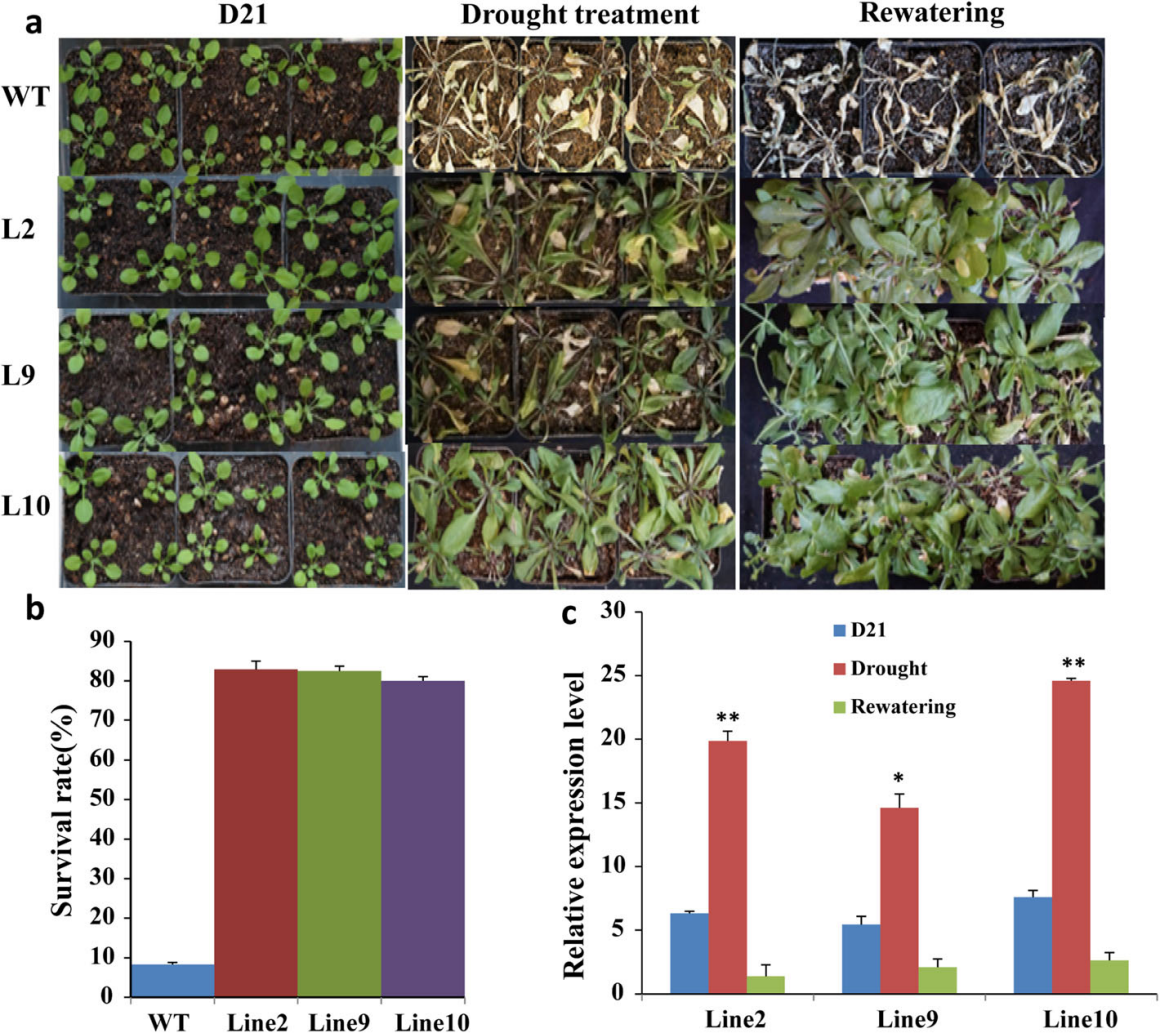
Performance of *CkWRKY33* transgenic Arabidopsis and wild-type (WT) plants under normal growth and drought-stress conditions. Three lines were randomly selected for the phenotypic screening. **a** Phenotypes of potted WT and transgenic plants after 21 d under normal growth, drought stress conditions and after rewatering; **b** Survival rates of WT and transgenic lines 2 d after re-watering. Each data point is the mean of three replicates of 20 plants. The error bars indicate the SD, Asterisks indicate statistical significance (*: *P* < 0.05; **: *P* < 0.01; Student’s t-test) of differences between transgenic lines and WT; **c** Expression levels of *CkWRKY33* in WT and CkWRKY33-transgenic Arabidopsis plants

In addition to the mannitol-stress treatment, the seeds of three transgenic and WT lines were sown in soil. After these plants had grown for 21 d under normal conditions, watering was stopped. After 15 d of the natural drought treatment, most leaves of WT plants had lost their green color and turned yellow, or even died. The transgenic plants showed slight yellowing and curling at the leaf tips and dehydration; however, they grew well and had a normal phenotype. After rehydration, WT plants showed complete wilting and dehydration, while the leaves of transgenic lines showed a low degree of atrophy and good growth. Compared with under drought-stress conditions, the leaves of transgenic plants became tender after 3 d of rehydration, indicating that transgenic plants were strongly resilient after rehydration (Fig. 5a).

The survival rate of WT was 8.33%, which was lower than any of the three transgenic lines. The survival rate of the three lines was 80% on average. Thus, the expression of the transformed *CkWRKY33* increased the drought resistance of transgenic plants (Fig. 5b).

A qRT-PCR experiment was used to analyze the expression patterns of *CkWRKY33* in transgenic plants before and after drought treatment. The relative expression level revealed that compared with before drought treatment, the *CkWRKY33* was highly induced expression by drought treatment in transgenic plants (Fig. 5c). After the drought treatment, the gene expressed 3 to 5 times more in transgenic plants than before drought treatment. After rehydration, the gene’s expression level in the transgenic plants decreased. Thus, that expression of the WRKY TF may improve the tolerance of transgenic Arabidopsis to drought stress.

### Changes in physiological traits under stress conditions

**Fig. 6:**
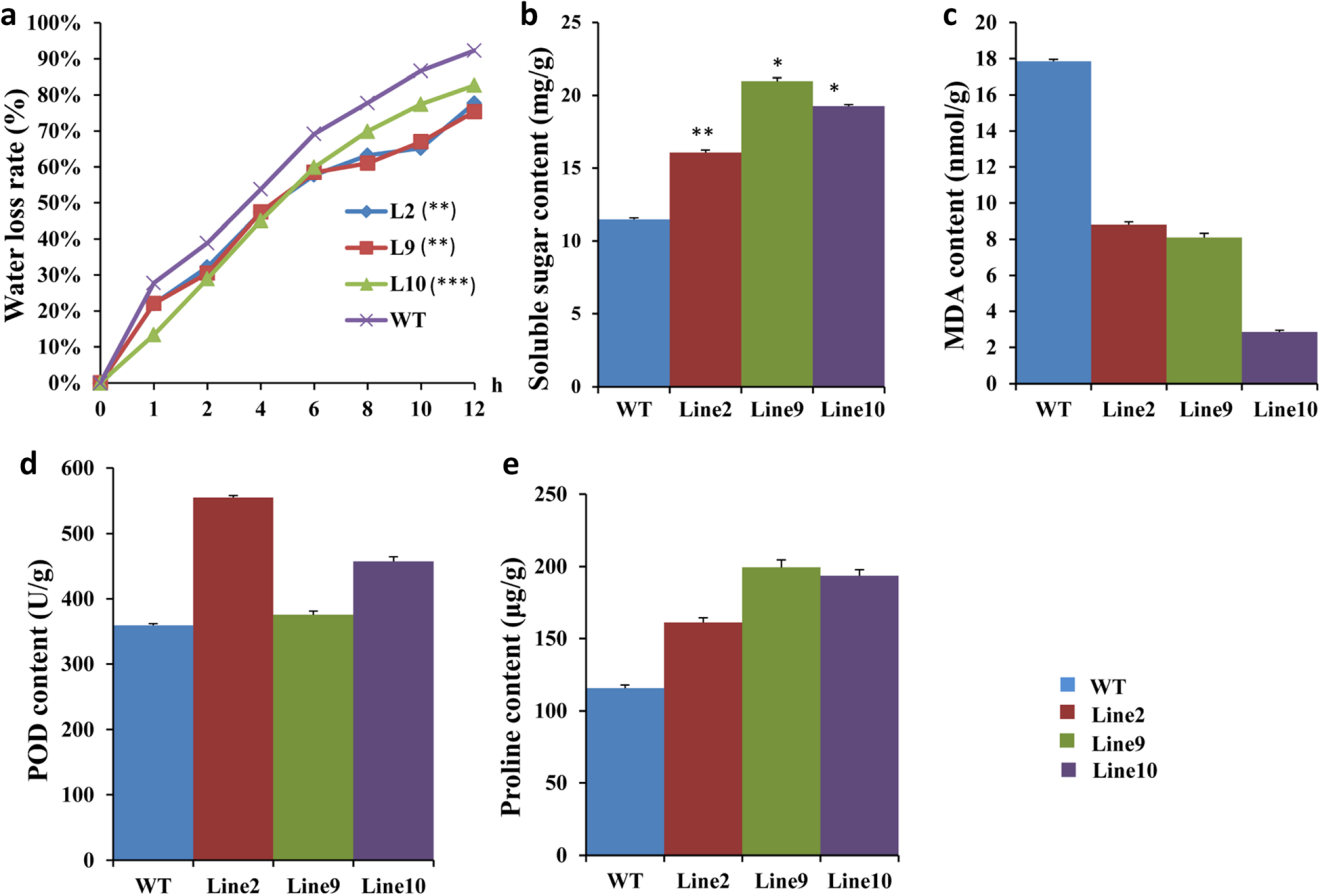
Physiological changes associated with drought-stress responses in wild-type (WT) and *CkWRKY33*-transgenic Arabidopsis plants. **a** Water loss rates of detached leaves from WT and transgenic plants. Each data point is the mean of three replicates of 10 detached leaves; **b** Soluble sugar content; **c** Malondialdehyde (MDA) content; **d** POD content; **e** proline content. All of the data values represent means±SD from three independent experiments. Asterisks indicate the statistical significance (*: *P* < 0.05, **: *P* < 0.01; Student’s t-test) of the differences between transgenic lines and WT plants

The leaf water loss rates of WT and transgenic plants were detected. The water loss rate increased as the processing time increased for all the plants. It was greater in WT than in transgenic plants (Fig. 6a). After 1 h, the water loss rate of the WT was 27.8% and the rates of the transgenic lines ranged from 11.8 to 20.5%. After a 12 h treatment, the water loss rates of all the transgenic lines were less than 86%, while that of the WT was as high as 92.3% (Fig. 6a). This indicated that the transgenic lines had lower water loss rates and stronger drought- tolerance levels.

The values of four physiological traits, soluble sugar content, malondialdehyde (MDA) content, proline content and peroxidase (POD) activity, were determined for the drought-treated transgenic plants. The soluble sugar content of each transgenic plant line was higher than that of the WT. The highest value for the transgenic plants was 23.70 mg/g and the lowest value was 16.08 mg/g. The MDA content was lower in transgenic plants than in WT, and the lowest value among the transgenic lines was 2.32 nmol/g. Thus, as the MDA content in the transgenic plants decreased, the damage to plant cell membranes decreased and the drought resistance increased. The proline content was higher in transgenic plants than in WT after drought stress, and the POD content followed the same trend (Fig. 6b-e).

## Discussion

WRKY TFs play important roles in plant responses to biotic and abiotic stresses. However, there is limited research regarding the gene functions of WRKY TFs in the desert tree *C. korshinskii* Kom. In this study, we isolated the *CkWRKY33* gene from *C. korshinskii* Kom. The gene structure and evolutionary relationships were analyzed, and then, gene function was confirmed using a transgenic approach. The current study is important in elucidating WRKY protein-regulated responses to abiotic stress in *C. korshinskii* Kom..

According to the transcriptome assembly sequence, the CDS sequence of *CkWRKY33* was cloned. Based on the high similarity between the CkWRKY33 protein and other WRKY proteins obtained from Arabidopsis, *Cicer arietinum*, *Glycine max* and *Vigna angularis*, we confirmed that the gene isolated from *C. korshinskii* Kom. is a WRKY gene and that belonged to Group-I. The results of subcellular localization of 35 s-CkWRKY33-GFP showed that the GFP signal was located in the nucleus, which suggested that CkWRKY33 actually function in the nucleus. The phylogenetic analysis of the CkWRKY33 sequence, together with the orthologous WRKY TFs from different plant species, such as Arabidopsis, maize, and *Glycine max*, revealed a phylogenetic tree having two distinct clades (Fig. 2). WRKY33 has evolved substantially after the divergence of dicots and monocots from their last common ancestor [28].

Until now, there were some researches involving the functions of WRKY33 in different species, including in Arabidopsis [27], oilseed rape [29] and maize [30]. In Arabidopsis, the over-expression of *WRKY33* was sufficient to increase Arabidopsis NaCl tolerance, and the same function was found in maize. In oilseed rape, over-expression of *WRKY33* could increase the Sclerotinia resistance. To investigate the mechanisms by which CkWRKY33 confers abiotic stress tolerance, we performed several experiments to monitor the phenotypic and physiological changes associated with drought responses. Mannitol is a sugar alcohol that is associated with plant stress resistance and is found in bacteria, fungi, and many higher plants [31, 32]. In this study, it was used to simulate natural drought-treatment conditions. Using mannitol stress, the phenotypic and root length changes of the transgenic plants could be observed. Thus, this provided an indicator system for the identification of drought resistance in *C. korshinskii* Kom..

POD activity, as well as soluble sugar, proline and MDA contents, are generally important physiological indicators of stress resistance in plants [33, 34]. In this study, soluble sugar, MDA and proline contents, as well as the POD activity, in WT and transgenic plants were determined under drought-stress conditions. Soluble sugar has a strong hydration capability, and its content increases under stress, which aids cells in holding water and preventing further damage [35]. The soluble sugar contents in the transgenic plants were greater than in that in WT plants after exposure to drought stress. In addition to the soluble sugar contents, the proline contents in the transgenic plants were also greater than that in WT plants after exposure to drought stress. The result was consistent with a previous study [36]. As a member of the antioxidant enzyme defense system, a high POD activity can reduce the accumulation of ROS, weaken the damage to cells and improve the stress resistance of plants. In this study, after drought-stress exposure, the POD activity levels in transgenic plants were greater than in WT plants. This was also found in other species. For example, *TaWRKY10* overexpression enhances drought stress, which may be caused by the decrease in ROS accumulation in tobacco [37]. MDA is a product of membrane lipid peroxidation, and its content is used to evaluate the tolerance of plants under stress conditions. Thus, it is used as a marker of lipid peroxidation and, therefore, of membrane damage [38]. We observed a lower MDA content in 35S::CkWRKY33 transgenic seedlings than in WT after exposure to drought stress. In conclusion, these analyses suggested that the over-expression of the *CkWRKY33* gene in Arabidopsis increased the content of these substances, which may result in improved drought tolerance in transgenic plants.

## Conclusion

In this study, a WRKY transcription factor, *WRKY33* was cloned from the desert tree, *Caragana korshinskii*, which has the characters of high tolerance to abiotic stress. Gene structural analysis showed that it belonged to the Group-I type. Subcellular localization experiments showed the presence of CkWRKY33 in the nucleus. Then *CkWRKY33* was over-expressed in the model plant Arabidopsis. When the over-expressed transgenic plants and WT were treated with drought stress, the transgenic lines showed higher survival rates, as well as relative soluble sugar, proline and peroxidase contents, but lower malondialdehyde contents. All the results mean that CkWRKY33 may act as a positive regulator involved in the drought-stress responses in *Caragana korshinskii*.

## Methods

### Plant materials

The seeds of *C. korshinskii* Kom. (http://www.iplant.cn/info/Caragana%20korshinskii?t=foc) were collected by Dr. Xinwu Pei from the Minqin Shasheng Botanical Garden in Gansu Province, China. The seeds were sown in a greenhouse and used as a source of material to clone *WRKY33*. *A. thaliana* ecotype Columbia-0 was used for the overexpression experiments and the WT and transgenic *A. thaliana* lines were grown at 22 °C, 70% relative humidity and a long-day photoperiod (16-h light/8-h dark).

### *CkWRKY33* cloning and sequence analysis

Total RNA was extracted from the leaves using the ZR Plant RNA MiniPrep Kit (ZYMO RESEARCH, Beijing, China), following the manufacturer’s protocol. First-strand cDNA was synthesized using PrimeScript™ RTase (TaKaRa Biotechnology, Dalian, China) according to the manufacturer’s instructions. A *CkWRKY33* cDNA corresponding to the predicted ORF was amplified by PCR using the gene-specific primers F1 (5′-ATGACTATGGATGATCATAACTG-3′) and R1 (5′-TTAGAAGTCCTTTGACATAAAT-3′). The PCR product was cloned into the pEasy-T1 cloning vector (Transgen, Beijing, China), and was then sequenced. Amino acid sequences of homologous WRKY33 proteins from other plant species were obtained from the NCBI database (https://www.ncbi.nlm.nih.gov)using BLASTP. A multiple sequence alignment of the deduced protein sequences and phylogenetic analyses were carried out using the DNAMAN software.

### Subcellular localization of CkWRKY33

Using in-fusion homologous recombination technology, the CkWRKY33 full-length DNA sequence fragment was inserted into a CaMV 35 s-GFP vector constructed previously by our laboratory to obtain the recombinant fusion construct 35 s-CkWRKY33-GFP. Then the new recombinant vector (35 s-CkWRKY33-GFP) and the control (35 s-GFP) were independently delivered to competent cells of *Agrobacterium tumefaciens* LBA4404 using the freeze-thaw approach. After the positive bacterial clones were identified, yeast extract peptone medium was employed to cultivate these clones, as well as the mRFP-AHL22 strain conserved by the laboratory as a localization marker [39]. The medium was supplemented with the appropriate antibiotics, 5 mM MES (Ph = 5.7) and 200 μM acetosyringone. When the bacterial solution’s concentrations reached OD600 = 0.6–1.0, they were centrifuged at 8000 rpm for 6 min to harvest the bacterial sediment. The sediment was washed with buffer containing 10 mM MgCl_2_, 10 mM MES twice and resuspended in the buffer above supplemented with 200 μM acetosyringone. The suspension’s concentrations were adjusted to OD600 = 0.5–0.6, and then, it was placed at 4 °C in the darkness for 3–4 h. Before injecting the *Nicotiana benthamiana* leaves, the suspension of mRFP-AHL22 was added at a 1:1 ratio and mixed well. The mixture was infiltrated into tobacco leaves using a syringe. The GFP signals in leaves were observed under a laser scanning confocal microscope after 24–48 h.

### Generation of transgenic *A. thaliana* plants over-expressing the *CkWRKY33* gene

The coding sequence of *CkWRKY33* (with EcoRI and XmaI sites added to its 5′ and 3′ ends, respectively) was amplified from pEasy-T1-CkWRKY33 using gene-specific primers F2 (5′-ACTGACGTAAGGGATGACGCACA ATGACTATGGATGATCATAACTG-3′) and R2 (5′-GTTGCTAGCACTATTGCCAAAAA TTAGAAGTCCTTTGACATAAAT-3′). It was then inserted in the plant over-expression vector, 35sRED using the in-fusion method, and called 35S::CkWRKY33. *A. tumefaciens* EHA105 harboring the 35S::CkWRKY33 construct was used to transform Arabidopsis by the floral-dip method [40]. T_0_ seeds were harvested and then the positive transgenic seeds were selected using hand-held green fluorescent flashlight through a red filter in the dark. If the seeds with red fluorescence were observed, the seeds were confirmed as the positive transgenic seeds. Then these positive seeds were sowed and self-pollinated until the T_2_ generation. Finally, the T_2_ homozygous lines were generated and used for all the subsequent experiments.

### Drought stress treatments of transgenic *A. thaliana* lines

To test the effects of drought stresses, 5-d-old transgenic and WT seedlings grown on 1/2MS medium plates were transferred to plates containing 1/2MS medium, or 1/2MS medium supplemented with either 50 mM or 100 mM mannitol. WT and transgenic Arabidopsis seeds were planted in cultivation pots at a density of four seeds per pot, using a total of 24 seeds. Three replicates were set and cultured in a greenhouse under 16 h light / 8 h dark conditions. After 3 weeks of plant growth, a natural drought treatment was carried out. WT plants were used as the controls. After the WT plants showed signs of death, all the plants were rehydrated for 2–3 d to determine their survival rates and the phenotypes of transgenic and WT plants were recorded.

To determine the water loss rate, 10 leaves were detached from 4-week-old transgenic and WT plants and immediately weighed. The samples were then placed on dry filter paper at a relative humidity of 40–45% at room temperature and weighed over a time course. The water loss rate was calculated as previously described [41].

### Gene expression analysis by quantitative real-time RT-PCR

Samples were taken from 3-week-old WT and transgenic *A. thaliana* seedlings after 15 d of drought treatment and 3 d of rehydration.

Total RNA was extracted from Arabidopsis leaves using an RNA prep plant kit (Tiangen Biotech.,Beijing, China) following the manufacturer’s protocols. First-strand cDNA was synthesized using PrimeScript™RTase (TaKaRa Biotechnology,Beijing, China) according to the manufacturer’s instructions. The quantitative real-time RT-PCR (qRT-PCR) analysis was conducted using SYBR green (TaKaRa Biotechnology) and an ABI7500 real-time RT-PCR instrument with the following thermal profile: 95 °C for 30 s, 40 cycles of 95 °C for 5 s, and 60 °C for 30 s. Each reaction was performed in triplicate for each of the three biologically replicated sets of cDNA samples. To perform the melt-curve analysis, the following program was added after the 40 PCR cycles: 95 °C for 15 s, followed by a constant increase from 60 °C to 95 °C. *A. thaliana* Actin 1 (TAIR: *AT2G37620*, https://www.arabidopsis.org/servlets/TairObject?id=31592&type=locus) was used as the reference gene. Primers used for qRT-PCR are listed in Additional file 1. Relative gene expression values were determined by using the 2^-ΔΔCt^ method [42] The experiment was repeated three times.

### Measurements of the soluble sugar, MDA and proline contents and POD activity levels

The values of four physiological traits, soluble sugar content, MDA content, proline content and POD activity level, were determined for the drought-treated transgenic plants. Arabidopsis leaves were collected during the drought treatment. Each trait was determined using the appropriate kit, following the manufacturer’s instructions (Solarbio, Beijing, China).

## Supplementary Information

**Additional file 1.** Sequence of primers for gene cloning

## Data Availability

The sequence information of *CkWRKY33* gene can be found in the *Caragana korshinskii* RNA-seq data with NCBI website (https://www.ncbi.nlm.nih.gov/biosample/3121496). The datasets used and/or analyzed during the current study available from the corresponding author on reasonable request.

## Abbreviations

*A. thaliana*: *Arabidopsis thaliana*
*C. korshinskii* Kom.: *Caragana korshinskii* Kom.
CDS: Coding sequence
MDA: Malonyl dialdehyde
ORF: Open reading frame
POD: Peroxidase
qRT-PCR: Quantitative real-time PCR
WT: Wild-type

## References

[1] Zhu J (2016). Abiotic stress signaling and responses in plants. Cell.

[2] Seki M, Kamei A, Yamaguchi-Shinozaki K, Shinozaki K (2003). Molecular responses to drought, salinity and frost: common and different paths for plant protection. Curr Opin Biotechnol.

[3] Chen WJ, Zhu T (2004). Networks of transcription factors with roles in environmental stress response. Trends Plant Sci.

[4] Yamamoto M (2000). Study of the transcription factor function in vivo: an overview. Tanpakushitsu Kakusan Koso.

[5] Eulgem T, Rushton PJ, Robatzek S, Somssich IE (2000). The WRKY superfamily of plant transcription factors. Trends Plant Sci.

[6] Ulker B, Somssich IE (2004). WRKY transcription factors: from DNA binding towards biological function. Curr Opin Plant Biol.

[7] Zhang Y, Wang L (2005). The WRKY transcription factor superfamily: its origin in eukaryotes and expansion in plants. BMC Evol Biol.

[8] Sahebi M, Hanafi MM, Rafii MY, Mahmud TMM, Azizi P, Osman M (2018). Improvement of drought tolerance in rice (*Oryza sativa* L.): genetics, genomic tools, and the WRKY gene family. Biomed Res Int.

[9] Ling J, Jiang WJ, Zhang Y, Yu HJ, Mao ZC, Gu XF, et al. Genome-wide analysis of WRKY gene family in *Cucumis sativus*. BMC Genomics. 2011;12:471. 10.1186/1471-2164-12-471 PMC3191544 21955985

[10] He HS, Dong Q, Shao YH, Jiang HY, Zhu SW, Cheng BJ (2012). Genome-wide survey and characterization of the WRKY gene family in *Populus trichocarpa*. Plant Cell Rep.

[11] Zhao J, Zhang XM, Guo RR, Wang YQ, Guo CL, Li Z (2018). Over-expression of a grape WRKY transcription factor gene, VlWRKY48, in *Arabidopsis thaliana* increases disease resistance and drought stress tolerance. Plant Cell Tiss Org.

[12] Xie T, Chen CJ, Li CH, Liu JR, Liu CY, He YH. Genome-wide investigation of WRKY gene family in pineapple: evolution and expression profiles during development and stress. BMC Genomics. 2018;19:490. 10.1186/s12864-018-4880-x PMC6019807 29940851

[13] Li J, Besseau S, Toronen P, Sipari N, Kollist H, Holm L (2013). Defense-related transcription factors WRKY70 and WRKY54 modulate osmotic stress tolerance by regulating stomatal aperture in Arabidopsis. New Phytol.

[14] Chen JN, Nolan TM, Ye HX, Zhang MC, Tong HN, Xin PY (2017). Arabidopsis WRKY46, WRKY54, and WRKY70 transcription factors are involved in Brassinosteroid-regulated plant growth and drought responses. Plant Cell.

[15] Wu XL, Shiroto Y, Kishitani S, Ito Y, Toriyama K (2009). Enhanced heat and drought tolerance in transgenic rice seedlings overexpressing OsWRKY11 under the control of HSP101 promoter. Plant Cell Rep.

[16] Qiu YP, Yu DQ (2009). Over-expression of the stress-induced OsWRKY45 enhances disease resistance and drought tolerance in Arabidopsis. Environ Exp Bot.

[17] Song Y, Chen L, Zhang L, Yu D (2010). Overexpression of OsWRKY72 gene interferes in the abscisic acid signal and auxin transport pathway of Arabidopsis. J Biosci.

[18] Raineri J, Wang SH, Peleg Z, Blumwald E, Chan RL (2015). The rice transcription factor OsWRKY47 is a positive regulator of the response to water deficit stress. Plant Mol Biol.

[19] Okay S, Derelli E, Unver T (2014). Transcriptome-wide identification of bread wheat WRKY transcription factors in response to drought stress. Mol Gen Genomics.

[20] Fang XW, Li JH, Xiong YC, Xu DH, Fan XW, Li FM (2008). Responses of *Caragana korshinskii* Kom. To shoot removal: mechanisms underlying regrowth. Ecol Res.

[21] Wang X, Chen X, Liu Y, Gao H, Wang Z, Sun G (2011). CkDREB gene in *Caragana korshinskii* is involved in the regulation of stress response to multiple abiotic stresses as an AP2/EREBP transcription factor. Mol Biol Rep.

[22] Yang Q (2013). Cloning and expression analysis of CkLEA1 gene in *Caragana korshinskii* Kom. China Biotechnol.

[23] Yang QY (2013). Construction of a suppression subtractive hybridization library of *Caragana korshinskii* under drought stress and cloning of CkWRKY1 gene. Sci Silvae Sin.

[24] Ren AQ, Jin YI, Gao HW, Jun LI, Wang XM. Cloning and expression analysis of the promoter of *Caragana korshinskii* gene. Acta Pratacul Sin. 2013;22(2):165–70.

[25] Long Y, Wang Y, Wu S, Wang J, Tian X, Pei X (2015). De novo assembly of transcriptome sequencing in *Caragana korshinskii* Kom. and characterization of EST-SSR markers. PLoS One.

[26] Liu F, Li X, Wang M, Wen J, Yi B, Shen J (2017). Interactions of WRKY15 and WRKY33 transcription factors and their roles in the resistance of oilseed rape to Sclerotinia infection. Plant Biotechnol J.

[27] Jiang Y, Deyholos M (2009). Functional characterization of Arabidopsis NaCl-inducible WRKY25 and WRKY33 transcription factors in abiotic stresses. Plant Mol Biol.

[28] Zhang L, Zhao G, Xia C, Jia J, Liu X, Kong X (2012). A wheat R2R3-MYB gene, TaMYB30-B, improves drought stress tolerance in transgenic Arabidopsis. J Exp Bot.

[29] Liu F, Li XX, Wang MR, Wen J, Yi B, Shen JX (2018). Interactions of WRKY15 and WRKY33 transcription factors and their roles in the resistance of oilseed rape to Sclerotinia infection. Plant Biotechnol J.

[30] Li H, Gao Y, Xu H, Dai Y, Deng DQ, Chen JM (2013). ZmWRKY33, a WRKY maize transcription factor conferring enhanced salt stress tolerances in Arabidopsis. Plant Growth Regul.

[31] Ben Rejeb K, Lefebvre-De Vos D, Le Disquet I, Leprince AS, Bordenave M, Maldiney R (2015). Hydrogen peroxide produced by NADPH oxidases increases proline accumulation during salt or mannitol stress in *Arabidopsis thaliana*. New Phytol.

[32] Ruijter GJG, Bax M, Patel H, Flitter SJ, van de Vondervoort PJI, de Vries RP (2003). Mannitol is required for stress tolerance in Aspergillus Niger conidiospores. Eukaryot Cell.

[33] Cai RH, Zhao Y, Wang YF, Lin YX, Peng XJ, Li Q (2014). Overexpression of a maize WRKY58 gene enhances drought and salt tolerance in transgenic rice. Plant Cell Tiss Org.

[34] Guo R, Qiao H, Zhao J, Wang X, Tu M, Guo C (2018). The grape VlWRKY3 gene promotes abiotic and biotic stress tolerance in transgenic *Arabidopsis thaliana*. Front Plant Sci.

[35] Lilin Y, Zongping P, Jing K, Yongcheng T, Lingxiao P (2013). Drought resistance of four plant species in ecological regeneration on mining area under drought stress. Northern Horticulture.

[36] Verbruggen N, Hermans C (2008). Proline accumulation in plants: a review. Amino Acids.

[37] Wang C, Deng P, Chen L, Wang X, Ma H, Hu W (2013). A wheat WRKY transcription factor TaWRKY10 confers tolerance to multiple abiotic stresses in transgenic tobacco. PLoS One.

[38] Wang Y, Gao C, Liang Y, Wang C, Yang C, Liu G (2010). A novel bZIP gene from Tamarix hispida mediates physiological responses to salt stress in tobacco plants. J Plant Physiol.

[39] Wang X, Fan C, Zhang X, Zhu J, Fu YF (2013). BioVector, a flexible system for gene specific-expression in plants. BMC Plant Biol.

[40] Clough SJ, Bent AF (1998). Floral dip: a simplified method for agrobacterium-mediated transformation of *Arabidopsis thaliana*. Plant J.

[41] Guo RR, Zhao J, Wang XH, Guo CL, Li Z, Wang YJ (2015). Constitutive expression of a grape aspartic protease gene in transgenic Arabidopsis confers osmotic stress tolerance. Plant Cell Tiss Org.

[42] Quail MA, Kozarewa I, Smith F, Scally A, Stephens PJ, Durbin R (2008). A large genome center's improvements to the Illumina sequencing system. Nat Methods.

