# Supplementary material for: Enhanced tolerance to drought stress resulting from Caragana korshinskii CkWRKY33 in transgenic Arabidopsis thaliana
Source: BMC Genom Data. 2021 Mar 10;22:11. doi: 10.1186/s12863-021-00965-4 (PMC7945665; doi:10.1186/s12863-021-00965-4)
Supplement: Supplementary file 1 — Additional file 1. Sequence of primers for gene cloning [file 12863_2021_965_MOESM1_ESM.docx]

Additional file 1 Sequence of primers for gene cloning

| Name | Forward primer(5’-3’) | Reverse primer(5’-3’) |
| --- | --- | --- |
| Gene cloning | |  |
| *CkWRKY33* | ATGACTATGGATGATCATAACTG | TTAGAAGTCCTTTGACATAAAT |
| Gene expression analysis | |  |
| *CkWRKY33* | ACCATGGAAGGAAGAAGCGG | ACCATGGAAGGAAGAAGCGG |
| *AtActin* | GATGTTCAGGCGAGTGAGTGAG | CGTAGTCACCTTCTTCATCCGC |
|  |  |  |
